# Differences in the Efficiency of Cognitive Control across Young Adulthood: An ERP Perspective

**DOI:** 10.3390/brainsci14040347

**Published:** 2024-03-31

**Authors:** Martina Knežević

**Affiliations:** Department of Psychology, Catholic University of Croatia, Ilica 242, 10000 Zagreb, Croatia; martina.knezevic@unicath.hr

**Keywords:** young adulthood, cognitive efficiency, protracted development, ERP, word categorization, early 20s

## Abstract

Young adulthood is a period of major life changes when everyday life becomes much more complex compared to adolescence. Such changes require highly efficient cognitive control. Developmental studies show that structural changes in the brain areas that support complex behavior continue into the early 20s. However, despite the fact that at the beginning of young adulthood, important behavioral and brain restructuring still occurs, most studies use broad age ranges for young adults (from 18 to 40 years of age) as a reference point for “adult” behavior. The aim of this study was to investigate age-related differences in the efficiency of cognitive control across young adulthood. In total, 107 individuals participated in this study and were divided into three age groups: 19–21, 23–26, and 28–44. We used a visual word categorization task to assess cognitive efficiency and event-related potentials (ERPs) to track events that take place from the stimulus onset until the actual behavioral response. We found age differences in both performance and amplitudes of the ERP components during the early stages of processing — P2 and N2. Our findings provide important evidence for the continuation of age-related changes in brain dynamics that underlie the efficiency of cognitive control even in the early 20s.

## 1. Introduction

Young adulthood is a period of major life changes, when along with becoming of age come real-life challenges, such as completing education, entering the labor market, perhaps leaving the parental home, and starting a family [1]. These changes necessitate adjustments in personal goals and aspirations, such as developing priorities related to education, career, finances, family, friends, romantic partners, community, etc. Managing everyday duties becomes more complex compared to adolescence and includes planning and carrying out many short-term (e.g., putting together a day’s outfit) or long-term (adding to a savings account for an apartment/house deposit) activities [2]. Such unexpected or dynamic changes in everyday life require a high level of cognitive control [3]. Cognitive control refers to the ability to intentionally coordinate and select behaviors, emotions, and thoughts based on current demands and context while suppressing inappropriate or habitual actions [4].

Maturational brain changes that protract into the early 20s include changes throughout different regions of the brain. Researchers found thinning of the lobes (reductions in gray matter) throughout the cortex, particularly in frontal and temporal regions and basal ganglia [5,6], with evidence that temporal regions develop last. It is believed that this process is a result of synaptic pruning (elimination of unused synapses), which, in turn, increases the efficiency of neuronal circuits. These brain systems together support complex behavior, such as cognitive control [4]. Myelination—the insulation of axons that speeds the neuronal transmission in frontal, parietal, and temporal brain regions—also continues after adolescence. The result of protracted myelination is the establishment of mature connectivity that supports the functional integration of brain circuits known to underpin the refinement of cognitive control processes [7,8]. In sum, histological and neuroimaging studies of structural brain development show that changes in the brain areas that underlie cognitive control continue into the early 20s.

Even though cognitive control abilities (e.g., response inhibition, working memory, cognitive flexibility) are present already in childhood, in line with the structural brain refinement, these abilities undergo substantial improvements throughout adolescence and into early young adulthood [7,9,10]. While preceding developmental stages involve the procurement of skills and abilities that significantly change behavior, in young adulthood, improvements are at the plateau stage, and young adults should have reached stable levels of adult behavior. However, while much of the behavior already appears adult-like in adolescence, studies reveal evidence showing inefficient cognitive control even in early young adulthood. In one of our previous studies [11], using functional imaging (fMRI), we found differences in the neural basis of performance monitoring between two groups of young adults: those aged 18–19 and those aged 23–25. Participants aged 18–19 recruited the right inferior frontal gyrus (IFG) more than participants aged 23–25 during the performance monitoring, which refers to the ability to detect and correct errors. These differences in the right IFG activation during performance monitoring might reflect greater effort, less neural efficiency, or inappropriate distribution of resources in the early 20s [11]. Recent findings reveal the importance of the middle frontal gyrus for the inhibitory control network [12]. According to cognitive efficiency theories, individuals can perform cognitive operations in a different manner. Cognitive operations in typical adults are performed in a way that minimizes the allocation of resources important for the completion of the task without affecting the maximal performance [13]. This is one of the important markers of mature adult behavior.

To investigate the maturity of cognitive control in young adulthood further, we included a group of young adults aged above 28 years in the following studies. This is the age when we do not expect significant brain maturational changes anymore [1]. We used a Go/No-Go task that taps into response inhibition, one of the key cognitive control functions related to voluntary behavioral control, and event-related potentials (ERPs) to precisely (within the range of a millisecond) track the temporal flow of information in the brain [10]. Participants in their early 20s showed more impulsive responses compared to those in their early 30s, and these behavioral differences were accompanied by differences in ERP components–P2 reflecting stimuli evaluation and performance optimization, N2 reflecting attentional control and behavioral adjustment, and P3 reflecting response inhibition. When we analyzed differences in error processing [14], we found that young adults in their early 20s did not show post-error adjustments after impulsive errors, unlike young adults in their early 30s. We also found differences in error-related negativity (ERN)—an ERP marker of error detection—and error positivity (Pe)—an ERP marker of error awareness. Our findings confirmed that a mature level of efficiently functioning cognitive control, at least in the aspect of response inhibition, is still not reached in the early 20s.

Taken together, studies show protracted structural and functional maturation of various brain regions concomitant with changes in cognitive control abilities throughout adolescence and into the early 20s. Still, most studies use broad age ranges for young adults (from 18 to 40 years of age) as a reference point for “adult” behavior. It seems that at the beginning of young adulthood, important behavioral and brain changes that have not been found at the end of young adulthood still occur, pointing to immaturities, which may limit adult-like behavior in the early 20s. At this age, young adults are faced with significant challenges related to new responsibilities and obligations, and success or failure may set the course that will strongly affect the path of their adult lives. On the other hand, many mental disorders, such as depression or generalized anxiety disorder, are most prevalent between the ages of 18 and 25 [15,16], which makes the early 20s a period of vulnerability for psychological disorders.

The aim of the current study was to investigate age-related differences in the efficiency of cognitive control in young adulthood using a visual word categorization task and ERPs. Since cognitive control has been thoroughly studied in childhood and adolescence, to acquire a complete picture of its developmental advancements, we included two groups of young adults (aged 19–21 and 23–26) and compared them to adults aged above 28. These transitional stages of development from adolescence to full adult maturity have not received appropriate attention in the literature thus far, and this is one of the contributions of this study. We focused on ERPs due to their excellent temporal resolution to better understand the possible changes in the timing of neural processes underlying cognitive control in young adulthood. It was previously found that different levels of visual word processing can be used as experimental paradigms to measure the efficiency of cognitive control [17,18]. Words units that carry many of the interesting codes of analysis (e.g., orthographic or semantic) and processing distinctions (e.g., automatic vs. attentional) are relatively well-defined. Although performance accuracy and reaction times give us the end result of the whole processing system, ERPs provide continuous information about the events in-between that have led to the end result. The most known ERP component related to word processing is the N400 [19]. The N400 is a negative-going ERP component that peaks around 400 ms after word presentation and is related to processing the meaning of the word. Studies also identified two earlier components important for efficient word processing. The P2 is a positive-going component peaking between 150 and 300 ms after word presentation and is related to attentional processing in skilled readers [20]. The N2 is a subsequent negative-going component peaking between 200 and 350 ms after stimulus onset related to word recognition [21]. Since the N2 usually arises before the motor response, it has been linked to stimulus identification and classification through conscious attention [22]. In line with our previous studies and available literature, we expected to find age differences in ERP components, namely those reflecting early stages of processing—P2 and N2. As word categorization is a relatively easy task for typically developing young adults, we did not expect significant differences in performance.

## 2. Materials and Methods

### 2.1. Participants

One hundred seven participants (*N* = 107; 56 females) were included in the study, divided into three age groups: early 20s (19 females; average age *M* = 20, *SD* = 0.79; age range 19–21 years), mid 20s (19 females; average age *M* = 24, *SD* = 0.97; age range 23–26 years), and early 30s (18 females; average age *M* = 33, *SD* = 4.33; age range 28–44 years). Age grouping was based on our previous studies, which revealed differences in the neural basis of performance monitoring, response inhibition, and error processing between these age groups [10,11,14]. The number of participants in each group was determined based on the literature [23]. Five additional subjects took part, but two were excluded from the analysis due to excessive artifacts (more than 20% of artifacts from blinks and involuntary muscle contractions), while three did not finish the task due to technical problems. All participants were right-handed. None reported any mental illness or previous head injuries, and none used any medication at the time of the study. All had normal or corrected-to-normal vision, and were native Croatian speakers with no reported reading difficulties.

The participants were recruited on a volunteer basis via E-mails, social networking (Facebook), and advertisements at the University of Zagreb. The study conformed to the 1964 Declaration of Helsinki, the ethical standards of the American Psychological Association (APA) and was approved by the local Ethics Committee of the University of Zagreb (Class: 643-02/09-03/22, Reference number: 380-02/7-11-6, 30 November 2011). Participants were thoroughly familiarized with the laboratory settings, experimental procedure [24], and the tasks prior to taking part in the study. They were informed of their right to withdraw from the study at any time, without any consequences. Participants gave written informed consent prior to taking part in the study. The principal investigator provided contact information (E-mail and mobile phone number) to the participants so that they could contact her in case of any additional questions or concerns.

### 2.2. Psychological Tests

Participants completed a series of standardized psychological tests and questionnaires.

Cognitive Nonverbal Test was used an estimate of logical reasoning through non-verbal g-factor IQ [25]. It consists of 40 items with four geometrical shapes in each, and participants’ task was to mark one shape that is significantly different from the other three. The total score is a number of correct responses within 15-min time limit.

The Letter Digit Substitution Test (LDST) was used as a measure of information processing speed. Participants’ task was to replace the randomized letters as quickly as possible with the appropriate digit indicated by the key. The key gives the numbers 1 to 9, each paired with a different letter from the alphabet beneath the key. Total score is the number of correct substitutions made in 60 s [26,27].

The Barratt Impulsiveness Scale (BIS) was used to measure impulsivity [28]. It is a 15-item, self-rating scale, and each item is rated on a 4-point Likert-type scale (1 = rarely/never, 4 = almost always). The total sum of scores represents the level of impulsivity.

The Eysenck Personality Questionnaire (EPQ) was used to assess psychoticism, extraversion, and neuroticism [29]. It contains 90 items to which participants respond with yes or no, and the result is calculated as the sum of scores of items pertaining to each personality trait separately.

### 2.3. The Task

Visual word categorization task was designed to elicit perceptual and semantic components of visual word processing [18]. During the perceptual portion of the task, participants were asked to make a decision about the case of the letters (uppercase or lowercase), and during the semantic portion of the task participants were asked to indicate whether the presented word refers to a living or a nonliving item. The task was divided into four blocks: two perceptual categorization blocks and two semantic categorization blocks, with 160 words in each block (320 words in total for each condition). Each condition contained 80 living and 80 non-living items, half written in uppercase and half in lowercase. The order of the blocks was counterbalanced across participants and groups, half performing the perceptual decision block first, and the other half the semantic decision block first.

The words were mono-, di-, or trisyllabic, chosen as high in frequency from the Croatian Frequency Dictionary [30], and equated for length across conditions. The average word length was 5 (±1) letters. Each word was presented in the center of a computer screen (font: Arial, 30 pt), yellow against the black background, until a response was given, with a maximum of 1.8 s, and a fixed interval of 1.5 s between two words. The task was programmed in E-prime 2.0 (Psychology Software Tools, Pittsburgh, PA, USA), and participants gave their responses pressing one of the two keys on the Serial Response Box (S-R Box) with either left (living/uppercase) or right (nonliving/lowercase) index fingers.

### 2.4. Procedure

This study was conducted at the Laboratory for Psycholinguistic Research at the University of Zagreb, Croatia. After volunteering for the study, participants received information letters with detailed descriptions of the research. They provided demographic information, medical history, and handedness information, and completed psychological tests and questionnaires. Each received a short training with a block of practice trials identical to the real task to explain the instructions and to ensure correct performance. After the training, participants were fitted with a 32-channel EEG cap (actiCAP, Brain Products GmbH, Munich, Germany). Two vertical and two horizontal electro-oculogram electrodes (VEOG and HEOG) were attached for recording eye saccades and blinks. They were seated comfortably in an office chair in front of a 24″ monitor (Samsung SyncMaster T220) at a normal viewing distance of approximately 80 cm within a sound attenuated, electrically shielded room. Before the onset of each block, instructions appeared on the screen including the details about the task and a reminder to respond as quickly and accurately as possible.

### 2.5. Data Recording and Analysis

The EEG was continuously recorded using a standard 32-channel actiCAP EEG cap connected to the Brain Vision recording system (version 1.03; Brain Products GmbH, Gilching, Germany). Blinks and vertical eye movements were recorded by means of two Ag/AgCl sintered electrodes placed above and below the right eye, while horizontal movements were recorded from two Ag/AgCl sintered electrodes placed at the outer canthus of each eye. FCz was used as a reference during recording, which started when electrical impendence had been reduced to less than 5 K-Ohms by light abrasion of the scalp. The data were recorded with a band pass of 0.01–100 Hz and sampling rate of 1 kHz.

BrainProducts Analyzer software package version 2.0 (Brain Products GmbH, Gilching, Germany) was used for EEG data processing. All electrodes were re-referenced to the average of right and left mastoids (TP9/TP10). Correct trials were baseline corrected with respect to −200 to 0 ms pre-stimulus period and segmented into epochs ranging from 200 ms before to 1600 ms after stimulus onset. The proportion of rejected epochs varied around 5% per participant mainly due to blinks. The remaining epochs were averaged for each participant. Artifact-free, averaged ERPs were obtained for 155 (±4) trials in the perceptual and 152 (±4) trials in the semantic condition. The components of interest were determined based on the inspection of the individual waveforms for each age group and in the reference to the literature [22,31,32]. Next, peak amplitude and latency was computed for the P2 from a 150 to 200 ms time window to explore early stages of attentional processing. Mean area amplitude for the N2 component was computed over a 200–350 ms interval and for the N400 component over a 350–500 ms interval for each participant and condition: perceptual (lowercase, uppercase) and semantic (living, nonliving).

### 2.6. Statistical Analyses

Gender was initially included in the analysis. Since it did not interact significantly with age, it was subsequently left out of the analysis. All statistical analyses were performed using SPSS version 23 for Windows (IBM SPSS Statistics for Windows, Armonk, NY, USA).

Univariate ANOVAs were performed for all psychological tests and questionnaires, performance accuracy (the percentage of correct responses), reaction time (corresponding to correct responses), and ERP components (P2, N2, N400) for each condition separately (perceptual, semantic) with age (Early 20s, Mid 20s, and Early 30s) as between-subject factors. We only report data for the central midline (Cz) electrode, since this is the electrode where the components of interest have typically been reported to be most prominent [19,33].

When appropriate, Tukey’s post-hoc test was applied to check for age differences. Effects sizes were calculated using partial eta squared (η_p_^2^), with 0.01–0.05 showing small effect size, 0.06–0.13 medium, and 0.14+ large effect size [34]. A significance level of *p* < 0.05 was adopted.

## 3. Results

### 3.1. Psychological Assessment

Participants were well-matched in terms of non-verbal IQ, speed of information processing and personality traits (Table 1). There were no significant age differences in non-verbal IQ, speed of information processing, impulsivity, extraversion, neuroticism, or psychoticism.

### 3.2. Behavioral Performance

**Figure 1:**
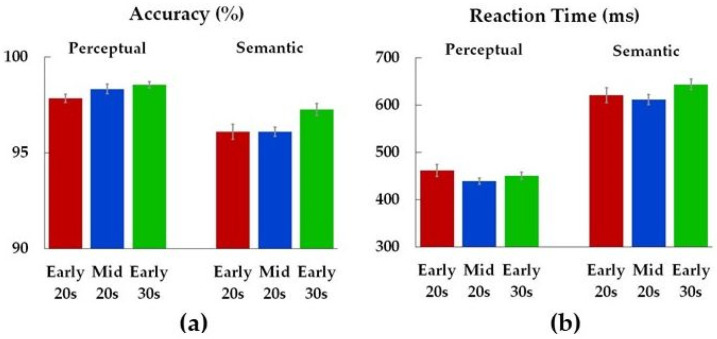
Accuracy (**a**) and reaction times (**b**) separate for each condition (perceptual, semantic) and age group (Early 20s, Mid 20s, Early 30s). Color bars represent means (*M*), with standard errors (*SE*) of the means on the error bars.

Age differences (Table 2) were found in performance accuracy during the semantic condition, with more correct responses (Figure 1a) in Early 30s compared to Early 20s (*p* = 0.04). There were no differences between Early 30s and Mid 20s (*p* = 0.83) or between Mid 20s and Early 20s (*p* = 0.13) in performance during the semantic condition. There were no other age differences (Table 2).

### 3.3. ERP Analysis

**Figure 2:**
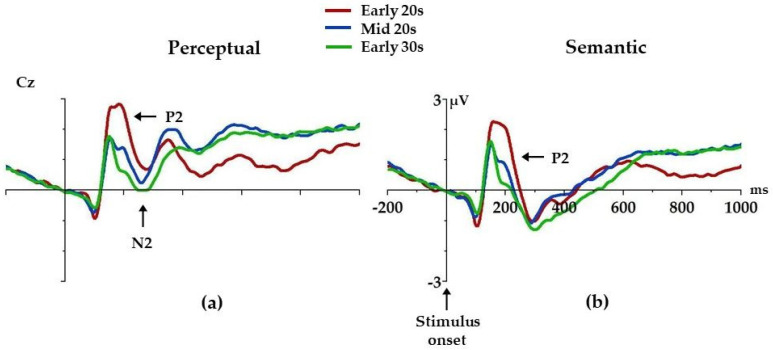
Grand average ERPs separate for each condition: perceptual (**a**) and semantic (**b**), and group (Early 20s, Mid 20s, Early 30s). Only components where age differences were found are marked with arrows. Positive is up.

Age differences were found for both perceptual and semantic P2 peak amplitudes and perceptual N2 mean amplitude (Figure 2, Table 3). Early 20s had higher perceptual P2 peak amplitude compared to both Mid 20s (*p* = 0.01) and Early 30s (*p* = 0.0002), with no differences in perceptual P2 peak amplitude between Mid 20s and Early 30s (*p* = 0.55). Similarly, Early 20s had higher semantic P2 peak amplitude compared to both Mid 20s (*p* = 0.047) and Early 30s (*p* = 0.0005), with no differences in semantic P2 peak amplitude between Mid 20s and Early 30s (*p* = 0.28). Moreover, Early 20s had higher perceptual N2 mean amplitude compared to Early 30s (*p* = 0.03), with no differences between Early 20s and Mid 20s (*p* = 0.92) or Mid 20s and Early 30s (*p* = 0.08).

## 4. Discussion

In this study, we aimed to investigate age-related differences in the efficiency of cognitive control in young adulthood. We used a visual word categorization task to assess cognitive efficiency and ERPs in order to track the events that take place from the stimulus onset until the actual behavioral response. In line with our hypothesis, we found differences in ERP components during early stages of processing. Early 20s had higher perceptual and semantic P2 peak amplitude compared to both Mid 20s and Early 30s, and higher perceptual N2 mean amplitude compared to Early 30s. Contrary to our expectations, we also found differences in the performance, where Early 20s made more errors during the semantic portion of the task compared to Early 30s.

The task used in this study had two conditions. During the perceptual condition, participants were asked to decide whether the presented word is written in uppercase or lowercase. During the semantic condition, participants were asked to decide whether the presented word refers to a living or a nonliving item. For skilled readers, such a task should be relatively effortless and easy to resolve. However, some level of attention and consideration is required to make a decision about semantic categorization, and during this semantic portion of the task, participants in their early 20s made more errors compared to participants in their early 30s. The literature identifies several reasons for performance differences in visual categorization tasks. Among the most cited possibilities are the effects of word frequency and age of acquisition. It has been found that words learned early in life, as well as those encountered frequently, are processed faster than words learned later in life or words encountered rarely [35,36]. The words chosen for the task used in this study were high in frequency [30], and we did not find age differences in the reaction times in any of the conditions. Considering that the average accuracy for all age groups was high (over 95%), we believe that it is highly unlikely that word frequency or age of acquisition would relate to the performance of Early 20s. It is more likely that performance differences in the semantic condition are reflection of differences in the efficiency of the attentional processing. The semantic decision is more complex than the perceptive decision, and involves several processing stages, including orthographic processing, attention, stimulus evaluation and categorization, and semantic processing [20]. Shortfalls in any of the processing stages may cause differences in performance. In our previous studies we found that even though young adults in their early 20s reached a high level of performance on response inhibition task, their performance was still not at the adult level and they made hasty errors [1,10,11,14]. As performance accuracy is the end result, ERPs can provide more detailed information about what happened between the stimulus onset and the response.

As expected, we found differences in ERP components reflecting the early stages of processing — P2 and N2. We know from previous studies that P2 reflects attentional processing and is enlarged when attention is paid to visual stimuli [20]. The N2 component has been found to reflect strategic monitoring and adjustment of motor responses [37]. Trials with more conflict elicit higher N2 amplitude, which is associated with the additional need to control response preparation [38]. Overall, such cognitive control mechanisms (attentional processing and conscious response monitoring) facilitate the processing of information that is most task-relevant, thus improving processing efficiency and preserving decision-making resources [39,40]. Since, for experienced readers, words are automatically processed and word recognition is a highly over-learned process, we speculate that this might be the source of differences in neural underpinnings of visual word categorization found in this study. Enlarged perceptual and semantic P2 indicate that participants in their early 20s invested more cognitive effort to process the presented words compared to participants in their early 30s. It seems that their ability to ignore irrelevant information (e.g., the meaning of the word in the perceptual condition) or additional information intake (case of the letters in the semantic condition) was less efficient, and they needed to invest more resources to complete this part of stimuli processing. Enhanced perceptual N2 might even reflect the additional need for control before the response is given due to the inappropriate distribution of cognitive resources. Hence, the presence of enhanced P2 and N2 components in the early 20s might be due to the insufficient higher-order control processes reflected in the strength of processes that focus attention on relevant stimuli in order to prevent a preoccupation with the irrelevant stimuli. Mature cognitive control enables individuals to resist habits or automatisms and to adapt to current situations. This is in line with cognitive efficiency theories, which presume that successful performance of cognitive operations includes investing minimal resources for maximal performance [3].

Previous ERP studies have found age-related decreases in the amplitude of the P2 component accompanied by behavioral performance improvements from childhood to adulthood [41,42]. Ladouceur, Dahl [43] compared early adolescents (around 12 years), late adolescents (around 16 years), and adults (around 29 years) using a flanker task and demonstrated that the N2 and the anterior cingulate cortex still mature during late adolescence, affecting the development of response monitoring processes. Pammer, Hansen [44] aimed to outline the spatiotemporal changes of cortical activity, which underlies word recognition, using magnetoencephalography (MEG). They found that the fusiform gyrus, known as the visual word form area, activates around 200 ms after stimulus onset and that this activity is preceded by the activity of the inferior frontal gyrus. Other functional imaging studies show activation of the left inferior frontal gyrus in the first 200 ms of reading and suggest very early interactions between the vision and language domains during visual word recognition [44,45,46]. The inferior frontal gyrus is one of the last brain regions to mature [9,47], and we previously found age-related differences in the right IFG activation during performance monitoring, possibly reflecting less neural efficiency or inappropriate distribution of resources in the early 20s.

The N400 is associated with a higher level of word processing system, elicited only by stimuli that allow deep (semantic) processing [19]. Indeed, developmental research suggests that the N400 is already relatively mature by late childhood [48], with subtle improvements during the school years [49]. Based on the results from our study, we speculate that the neural substrates giving rise to word recognition are matured and fully functional in the early 20s. However, attentional resources underlying the initial stages of visual word processing and response preparation are still rearranging at this age, which may hinder performance even in relatively undemanding cognitive tasks.

As mentioned in the introduction, most developmental studies use broad age ranges when referring to “adult” behavior without considering the protracted structural and functional brain maturation that occurs during the early 20s, which could have significant effects on performance. Our findings provide important evidence for the continuation of age-related changes in brain dynamics that underlie word processing even in the early 20s. It is possible that this life period reflects a transitional stage of attentional network refinement, possibly due to the slow maturation of cortical networks, which, while still rearranging, may not be recruited effectively. This is in line with the information processing theories, which emphasize that the advancements in various aspects of cognitive control are based on underlying biological refinements that allow progressive increases in processing capacities [50,51]. In the context of histological and structural MRI studies discussed in the introduction, it is possible that while synaptic pruning and increased myelination still occur during the early 20s, the cognitive control is less efficient and only after the interconnections of the association cortices are fully myelinated and excess synapses pruned is the adult performance is achieved. Since we used EEG to track the temporal flow of the information in the brain between stimulus presentation and response, new studies could benefit from including other brain imaging methods, such as fMRI, to identify brain regions implicated in developmental changes in cognitive control after adolescence. Future clinical and nonclinical developmental studies should consider including a narrow age range in young adult cohorts when investigating the developmental differences in the efficiency of cognitive control. We used a visual word categorization task as a measure of cognitive efficiency. Since this is a multifaceted construct, future studies could include other tasks that measure, for instance, processing speed or attention and vigilance, which would give important information about the development of cognitive efficiency.

## Figures and Tables

**Table 1:** Summary of statistics for psychological assessment.

	Early 20s *M* (±*SD*)	Mid 20s *M* (±*SD*)	Early 30s *M* (±*SD*)	*F_(2,104)_*	*p*	η_p_^2^
Non-verbal IQ	33.8 (±3.96)	31.7 (±5.19)	31.7 (±5.47)	2.1	0.13	0.04
Speed of info. processing	45.1 (±4.86)	46.0 (±5.55)	44.6 (±4.99)	0.7	0.49	0.01
Impulsivity	29.2 (±4.74)	28.9 (±5.60)	29.5 (±5.05)	0.2	0.86	0.003
Extraversion	14.4 (±4.94)	15.3 (±4.17)	14.4 (±4.36)	0.4	0.64	0.01
Psychoticism	3.5 (±2.45)	3.7 (±2.14)	4.4 (±2.02)	0.7	0.48	0.01
Neuroticism	8.9 (±4.58)	7.9 (±5.35)	6.8 (±4.35)	1.9	0.16	0.03

**Table 2:** Summary of ANOVAs for the behavioral performance.

Variable	*F*	*p*	η_p_^2^
*Accuracy*			
Perceptual	2.7	0.07	0.05
Semantic	3.5	0.04 *	0.06
*Reaction Time*			
Perceptual	1.4	0.24	0.03
Semantic	1.7	0.18	0.03

Note. * *p* < 0.05.

**Table 3:** Summary of ANOVAs for the ERPs.

Variable	*F_(2,104)_*	*p*	η_p_^2^
*P2 Peak Latency*			
Perceptual	3.1	0.05	0.06
Semantic	2.2	0.11	0.04
*P2 Peak Amplitude*			
Perceptual	9.4	<0.001 ***	0.15
Semantic	7.8	0.001 **	0.13
*N2 Mean Amplitude*			
Perceptual	3.8	0.03 *	0.07
Semantic	2.0	0.14	0.04
*N400 Mean Amplitude*			
Perceptual	3.2	0.05	0.06
Semantic	1.1	0.35	0.02

Note. * *p* < 0.05; ** *p* < 0.01: *** *p* < 0.001

## Data Availability

Research and analysis materials are available on request. The data are not publicly available due to privacy.
